# Nurses’ experiences of health concerns, teamwork, leadership and knowledge transfer during an Ebola outbreak in West Africa

**DOI:** 10.1002/nop2.258

**Published:** 2019-03-21

**Authors:** Jessica Holmgren, Stéphanie Paillard‐Borg, Panu Saaristo, Eva von Strauss

**Affiliations:** ^1^ The Swedish Red Cross University College (SRCUC) Huddinge Sweden; ^2^ The International Federation of the Red Cross and Red Crescent Societies (IFRC) Geneva Switzerland

**Keywords:** Ebola outbreak, global nursing, health concerns, knowledge transfer, leadership, nurses, teamwork

## Abstract

**Aim:**

To describe nurses’ experiences of health concerns, teamwork, leadership and management and knowledge transfer during an Ebola outbreak in West Africa.

**Design:**

The study has a qualitative descriptive design.

**Methods:**

The 44 nurses who had worked in an Ebola Treatment Centre in Kenema in 2014 and 2015 were invited by email to respond to a questionnaire. The qualitative, open‐ended answers were analysed using a thematic analysis. Data have been coded systematically, with the identification of semantic patterns presented in four themes.

**Results:**

The themes are as follows: personal health management—a way to feel safe and secure for delegates and affiliates; pre‐deployment training—crucial for a joint value base and future collaboration; the importance of a professional democratic approach and being a good role model; and the value of timely in‐depth knowledge transfer of experienced former delegates.

## 1 INTRODUCTION

The Ebola virus disease (EVD) is one of the deadliest diseases in the world. There are five different types of Ebola, and the disease is classified as a viral haemorrhagic fever. The EVD is named after a river in the Democratic Republic of Congo, where it was discovered for the first time in 1976. African countries have since suffered from Ebola epidemics to varying degrees. Uganda, Nigeria, Senegal and Mali have all reported Ebola outbreaks. However, Guinea, Sierra Leone and Liberia (all situated in West Africa) have been hit extremely hard, with 28, 616 sick people and 11,310 deaths by the summer of 2018. The EVD is believed to be transferred from fruit bats to humans. The deadly virus is then further transmitted between humans through bodily fluids such as blood, secretions, vomit and sweat. Many people have become infected by EVD after they have cared for and buried their sick relatives. After an incubation period of up to 3 weeks, people who are infected develop symptoms including high fever, headache and muscle pain. These symptoms are followed by a rash, diarrhoea and vomiting. As the illness progresses, the body is affected by organ failure and necrotic bleeding organs (Médecins Sans Frontières, 2018a). The fatality rate of the EVD has been as high as 90% in the African continent. Those who have fallen ill in the Western world have had a significantly greater chance of survival due to intensive care (Rubin & Baden, 2014). The latest Ebola outbreak, in the Democratic Republic of Congo, has been carefully monitored. So far, this specific EVD epidemic has claimed more than 160 lives (WHO, 2018a). To prevent further Ebola epidemics, efforts are currently focussed on control and surveillance based on five principles. These are as follows: isolation and care of patients in the established Ebola treatment centres (ETC); safe funerals; information; tracing of infection; and effective care for those who have not been infected by EVD (Médecins Sans Frontières, 2018b). The work focuses on teaching people in vulnerable and exposed areas how they can protect themselves from becoming infected with EVD (WHO, 2018b). After the largest outbreak of Ebola in West Africa, which posed a global threat to human health, attempts have been made to produce a vaccine that can protect against the virus (Médecins Sans Frontières, 2018b). Early trial results from pilot studies have shown positive outcomes Lancet (2015; WHO, 2016), although this is only the beginning of a long process to develop an approved vaccine. Since EVD is an insidious disease and health prevention and healthcare resources are limited in some parts of the world, there is always a risk of new Ebola outbreaks (Médecins Sans Frontières, 2018a, 2018b).

Nurses, among other healthcare workers, have a long tradition of helping and caring for people in disasters both locally and globally. Nurses worldwide strive to maintain health, relieve suffering and advocate for vulnerable persons to protect the health of present and future generations (Holmgren, 2017). In the 2014–2015 Ebola outbreak in West Africa, nurses played a crucial role as first responders during this global crisis. Worldwide organizations such as the World Health Organization (WHO), Médecins Sans Frontières (MSF), the International Federation of Red Cross and Red Crescent Societies (IFRC) and the Sierra Leone Red Cross (as well as local authorities) mobilized resources and cooperated to get an overview of what was required to bring the EVD outbreak under control. This required patience and the ability to complete demanding work, with sometimes‐scarce resources, under very difficult working conditions. Despite careful preparations and safety regulations being in place pre‐deployment, local and international healthcare workers have nonetheless lost their lives because they have been infected by Ebola (www.ifrc.org).

The IFRC is the largest humanitarian non‐governmental organization in the world. It has extensive experience of humanitarian work and acts quickly and efficiently in the event of major crises around the world. One of the organization's top priorities is to provide an adequate global disaster management system to respond to people's needs in times of disaster and crisis. The IFRC focuses on areas related to health and ill health, migration and risk reduction in the event of disasters. This complex work is often based on volunteer efforts in local communities. It involves millions of volunteers who belong to the organization. In major crises, such as the 2014–2015 Ebola outbreak in West Africa, international reinforcement was also needed. About 80 nurses volunteered to establish and operate an ETC in Kenema, Sierra Leone. It was the first time that the IFRC had sent healthcare workers to an Ebola outbreak. Although the IFRC has many years of experience in handling disasters, the Ebola outbreak presented a new challenge because of its complexity and unpredictability—not least for the local nurses who did not realize that outreach patients suffered from the highly infectious and lethal Ebola virus until it was too late (www.ifrc.org).

## 2 BACKGROUND

By examining previous research on EVD, nurses and nursing between 2014–2018, it is possible to gain an overview of the main areas previously investigated. Research questions have previously addressed:

- How healthcare workers are prepared to care for Ebola patients in West Africa (Adongo et al., 2017; Andertun, Hörnsten, & Hajdarevic, 2017; Annan et al., 2017; Cranmer et al., 2015; Gee & Skovdal, 2017a, 2017b; Kollie, Winslow, Pothier, & Gaede, 2017; Von Strauss, Paillard‐Borg, Holmgren, & Saaristo, 2017; Turtle et al., 2015).
- The importance of nurses’ skills and competences in fighting Ebola (Baltzell, McLemore, Shattell, & Rankin, 2017; Sagar, 2015).
- How healthcare workers/nurses are treated on their return after an Ebola mission (Gee & Skovdal, 2018).
- How nursing students are prepared to care for Ebola patients in the Western care context (Chilton, McNeil, & Alfred, 2016; McNiel & Elertson, 2017).
- How well‐prepared healthcare professionals/nurses in a Western context are to care for Ebola patients (Baduage, Moss, & Morphe, 2017; Eckes et al., 2016; Rajiah et al., 2016; Smit et al., 2017).
- Perceptions of healthcare workers/nurses who have been infected with Ebola (Johnson, 2016).
- Nurses’ attitudes towards caring for patients with Ebola in a Western care context (Narasimhuli et al., 2015; Speroni, Seibert, & Mallinson, 2015).
- The public's understanding of nurses’ caring role in an Ebola outbreak (McGillis Hall & Kashin, 2016).

In summary, previous research related to Ebola has focused on a range of different perspectives across local and global healthcare contexts. However, there seems to be a need for research that describes nurses’ own experiences of different aspects of fieldwork in the fight against EVD. Based on such knowledge, it would be possible to develop and improve strategies for preparing nurses to work in extreme care situations such as an Ebola outbreak. More specifically, the question we sought to address was the following: how do nurses experience issues related to their own health, leadership and necessary knowledge during a disaster? The aim of this study was to describe nurses’ experiences of health concerns, teamwork, leadership and management and knowledge transfer during the Ebola outbreak in West Africa.

## 3 METHOD

This study is part of a larger research project concerning global nursing during the Ebola Virus Disease outbreak in West Africa, before, during and after deployment of nurses in IFRC (Von Strauss et al., 2017). The study has a qualitative descriptive design.

### 3.1 Study context

Several ETCs were established in West Africa to care for people affected by Ebola. The IFRC's first ETC was built over a period of 3 weeks in Kenema, Sierra Leone. Initially, the centre was staffed by just under 20 international nurses and an even larger number of local nurses. All work was based on rigorous planning and preparation to ensure that no staff members were exposed to Ebola. This involved disciplined training in the use of the physical protection equipment that was worn when interacting with those who were ill. At the centre, there was room for a total of 60 patients who were provided with care 24 hr a day. The ETC was organized into three different areas addressing confirmed, probable and suspected cases of Ebola, respectively. Each staff member had a specific, important role in the running of the centre. In addition to caring, the workload included being responsible for water and sanitation, cooking, providing clean clothes and arranging safe funerals (www.ifrc.org, 2014).

### 3.2 Sample

The IFRC facilitated the selection procedure by providing a list of email addresses to the last author. Nurses who were deployed by the IFRC in the ETC in Kenema in 2014 and 2015 were systematically invited by email to participate in this study. This comprised a total of 78 nurses, of which 13 had email addresses that returned as non‐valid. This left 65 nurses, of whom it was possible to approach 50. 15 nurses never responded to the invitation and thus did not indicate whether they wanted to participate in the study or not. Out of the remaining 50 email invitations, 44 nurses gave their informed consent to participate. 36 of these were women, and the remainder were men. They were aged between 25–61, and 33 of them had previous experience of voluntary work on humanitarian missions. 26 of the nurses had undergone training before going to the ETC, and for the most part, this was the first time they had worked in Kenema. Most nurses came from Europe, while 10 came from Oceania, 5 from North America and 2 from Africa.

### 3.3 Data collection

The nurses were asked to answer a questionnaire, distributed to them by email, covering ten questions including associated subqueries (see Appendix A). The questions covered various aspects of global nursing, with the focus of this study relating to health concerns, teamwork, leadership and management and knowledge transfer. The questionnaire covered demographic data as well as questions for which the informants could leave qualitative open‐ended answers. The questionnaire was designed based on the issues of interest raised by the IRFC in collaboration with the researching authors. This was the first time the IFRC was involved in an Ebola mission, so there was an interest in understanding various aspects in the field. A first draft of the questionnaire was designed to be validated by a group of nurses with many years of experiences of humanitarian fieldwork in the Red Cross movement. Their feedback was mainly about reducing the number of questions and making them more concrete and less comprehensive. They also stated that the questions could be more open and less defined. After a few rounds of revisions, the group of nurses confirmed that the questionnaire was clear and relevant to the aim of this study.

### 3.4 Data analysis

The data set was analysed using a thematic analysis (Braun & Clarke, 2006). The analysis is characterized by a semantic approach, given the nature of the data set. The data were organized at a descriptive level focusing on semantic content and its patterns. Initially, all the responses in the questionnaires were gathered in an Excel sheet, in relation to how the questions were presented and answered. The questions covered health concerns (questions 2a–d), teamwork, principles and behaviour (5a–b), leadership and management (6a–c) and knowledge transfer (7a–b). The data were transferred to a Word document to produce a relevant set of data in relation to the aim of this study. Issues concerning stress (3a–b), social and cultural environment (4) and attitudes (8) will be presented in another paper. The remaining items (1 and 9–10) covered demographic data, while the items “conclusions” and “miscellaneous” were not relevant for this study.

The data set was read through as a whole by the first author (JH) to obtain an overall understanding of its semantic content. Initial ideas and associations gathered while reading were noted down. The data were read through once again, and interesting features were coded systematically, question by question. The codes were highlighted in different colours, and the whole data set was reviewed in this way. The semantic content related to a question could include several different codes. The codes where then grouped based on similarities and differences. The codes that had similar meanings were grouped and constituted potential themes. The codes were further checked in relation to the semantic content, and the themes were adjusted to cover the entire data set. The themes were then refined and definitively named. Finally, the analysis was presented in this paper and the themes were highlighted through selected extracts from the data set (Table 1).

**Table 1:** Example of the data analysis presented in data extracts, codes and themes

Data extracts	Codes	Themes
“Yes. Adequate because they provided with all the info and resources needed i.e. immunizations, emergency, medical kit, malaria prevention and knowledge. Could take contact anytime” (no 11). “Trusted that family would get info in case of need, overall very supportive” (no 3)	Ample information Health screening Malaria prophylaxis Vaccinations and medical kit Support from doctors, RC, experts, delegates with previous experiences from fieldwork Free to ask questions Risk and safety issues Practical training Felt secure	Personal health management—a way to feel safe and secure for delegates and affiliates
“Very important, you need a good understanding of how the team works together, what the expectations are and rules around behavior. This keeps everyone safe and working together towards the same goal” (no 19). “It's always good to be reminded of the importance of teamwork especially in such a tense working environment where your health also depends on the actions of others and vice versa” (no 47)	Same training leads to the same attitude Work in a team. Taking care of colleagues Strong spirit and strong bonds between staff Rules around behaviour Working together towards the same goal It is important since the situations in the field differ so much from daily life at home	Pre‐deployment training—crucial for a joint value base and future collaboration
“A democratic management style. It is very important to collaborate in those kind of missions. Therefor a good communication and information of staff is mandatory” (no 35). “Informative, communicative, clear structures, social skills and professional competence. Experience in foreign missions” (no 36)	Democratic leadership, awareness of what others are doing Advocate democracy, hear all voices, autocrat leadership Democratic management style Social skills, professional, experiences from foreign missions Working for the same goal Keeping up the morale of the team	The importance of a professional democratic approach and being a good role model
“I think that RC did it very well, the training and discussions with delegates, who had been already in Ebola mission” (no 46). “More in depth knowledge but more importantly given in a more timely manner. I got the bundle of the information on the day of departure which was too late” (no 11)	Up to date sitreps are excellent tools for knowledge transfer Discussions with delegates who had already been on Ebola mission Not enough knowledge about operation and procedures Not sufficiently deep enough Too superficial Information at the right time	The value of timely in‐depth knowledge transfer of experienced former delegates

The results were presented to all authors by the first author. They were discussed and revised regarding consistency and trustworthiness to ensure the accuracy and interpretation of the data set. Overall, the co‐authors shared the interpretation of the data. However, there were a few suggestions of more suitable concepts to use to make the result section more stringent in relation to the data. For example, under one of the thematic headings, the concept of “democratic” was added to “professional” since it reflected the data in a more nuanced way.

### 3.5 Ethical considerations

Since this study involves people, the Declaration of Helsinki has been followed (World Medical Association, 2013). This means that the authors have safeguarded the participants' right to autonomy, integrity and confidentiality. Every participant has given their informed consent and received the information that they can withdraw their participation at any time without giving reasons for doing so. For an individual participant not to be identified, the participants' real names have been decoded. An advisory opinion has also been obtained from the Regional Ethical Review Board in Sweden, which states that there were no obstacles to conduct the study as it does not involve human biological material or the handling of sensitive personal data (The Regional Ethical Review Board, 2018). Furthermore, the Code of Conduct of the Red Cross (www.ifrc.org, 1991) has been followed where it is applicable in a research context and the IFRC has approved the study to be conducted.

## 4 RESULTS

**Figure 1:**
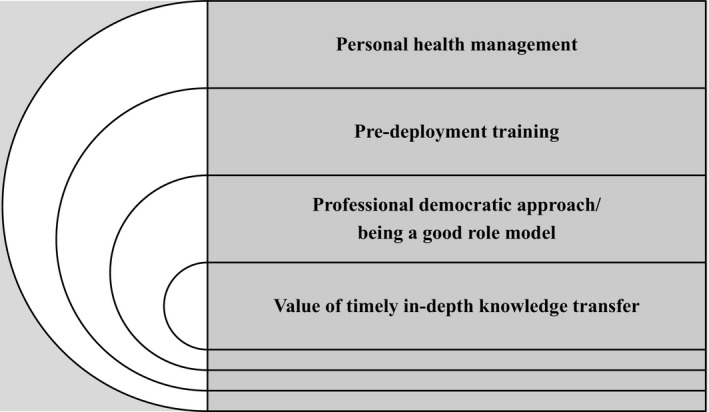
A summary of findings presented in four themes

The thematic analysis resulted in four themes emerging: Personal health management—a way to feel safe and secure for delegates and affiliates; pre‐deployment training—crucial for a joint value base and future collaboration; the importance of a professional democratic approach and being a good role model; and the value of timely in‐depth knowledge transfer by experienced former delegates (Figure 1).

### 4.1 Personal health management—a way to feel safe and secure for delegates and affiliates

Most of the participants experienced their personal health preparation, provided by IFRC, as adequate. This was expressed by one of the participants: “Superb preparation by Inter Health, pre and post health check as well as full medical kit and checking of our psychological wellbeing” (no 30). They stated that they had participated in pre‐employment courses where they received full information for the mission. The participants had also undergone medical health check‐ups and evaluation of mental well‐being, since the mission could be experienced as mentally stressful. As part of the preparation, they had received relevant vaccinations, malaria prophylaxis and reviews of the risks and safety issues that might appear in the field. Most participants felt that they got the support they needed from responsive nurses, doctors and former delegates. There was time to ask questions and practice which contributed to many of the participants feeling safe and secure in their role caring for Ebola patients.

However, a small number of the participants experienced a lack of information, unclear information and bad timing. One participant felt that s/he did not receive any information at all: “Do not remember having any health preparation from RC” (no 14)*,* and some other participants said that the information was given too late. One participant wished that more focus had been placed on psychosocial health, while another participant said that there was too much information about medical and psychological aspects. One participant said that s/he would have liked to have more information about the medication provided by the IFRC. Finally, some participants wanted more information about the return home and some of the participants felt that the IFRC needed to update them with acute information about the Ebola situation that prevailed.

Most participants found that the personal health follow‐up by the IFRC on the return of the mission was adequate. Participants described having clear and adequate instructions on what to do if they had any symptoms of Ebola infection after returning home. During the quarantine period, they received the support they needed, and they were convinced that they would have been taken care of quickly in the event of signs of illness. This is indicated in the following quotes: “Yes, they encouraged me to attend a psych debrief the day after returning. They kept in touch with quarantine up to 21 days” (no 11). Furthermore, the questionnaire shows that it was mandatory for participants to undergo medical and psychological health follow‐up via mobile phone. Participants also had the opportunity to receive support and assistance from nurses and former delegates as part of a debriefing strategy. One participant also shared that there was a number s/he could call in case of emergency, if s/he felt bad, physically or mentally. One of the participating nurses pointed out how important follow‐up was following a mission, in view of the stigma found in society around people who have been infected by Ebola. S/he expresses it thus: *“*It was very useful after this mission, because of the stigma in the society” (no 37). The health follow‐up therefore seems to fill several important functions.

Although most participants were satisfied with the personal health follow‐up, there were also some more critical views expressed in the questionnaire. These mostly related to perceived lack of information and late follow‐up. One participant had this experience: *“*Saw doctor only after quarantine time, not even a phone call to ask if all was ok” (no 33). Some nurses wanted more thorough information about the psychological help that was available. They also felt that the health checks were too basic, and they noted that no one had contacted them about their state of health at home. On the other hand, there were also those who felt that the health follow‐up was unnecessary or that the psychological follow‐up was not important or valuable.

Another important aspect of health management was whether the participants perceived that they received relevant information related to the mission and had the opportunity to voice their concerns. Most of them found that affiliates were well informed and offered the support of the IFRC that they needed. This is expressed in the following quote: *“*I found the information for family and friends to be a very useful tool. RC also offered to speak to any family or friends who had further questions for them as well as provide my family with a number to call if they needed support during my deployment” (no 39). The NGO thus encouraged affiliates to attend courses and followed up on any questions from them. However, some participants describe the opposite, arguing that affiliates did not receive any reliable information and that it was left up to them to inform and support loved ones. This is shown in this quote: “No, dealt with all concerns. Rules of behaviour upon return changed frequently, no adequate communication by RC. Bad information from RC regarding arrangement with resort for quarantine upon return” (no 24). In summary, most participants agree that the personal health management was important. However, there were aspects that could be improved, such as a more prompt and long‐term follow‐up. They also mentioned the need for a health‐responsible person at the IFRC, as well as for up to date guidelines and brochures focusing on health.

### 4.2 Pre‐deployment training—crucial for a joint value base and future collaboration

The second theme mirrors the nurses’ experiences of pre‐deployment training in teamwork, principles and behaviour. Most participants felt that the pre‐deployment training was important, valuable and filled a key role. The general experience was that the IFRC provided delegates with excellent training based on Red Cross principles and practical exercises. Nurses said that the training united all those who had a mission in Kenema, which led to a joint value base. A participant shares his/her experiences: *“*Very important, you need a good understanding of how the team works together, what the expectations are and rules around behaviour. This keeps everyone safe and working together towards the same goal” (no 19). Thanks to the training, they received an introduction to the context where they would be working and a foretaste of what awaited them in the field. Several participants also mentioned the importance of teamwork, as their own health and safety was linked to the actions of others. The participants also found that the training helped them to successfully cooperate with the local healthcare workers at the ETC and to work efficiently and helpfully in relation to the local population. Finally, it was valuable to get to know each other's experiences during the pre‐deployment training.

Although most participants were positive about the training, there was also a wish that the course had included fewer participants in each group. Some said that they did not receive any training on teamwork, principles and behaviour, which one participant described as necessary because the circumstances in Kenema differed so significantly from daily life at home. One participant had wanted more information about the Ebola infection, more authentic exercises and information on what one could expect in different positions in the field. Lastly, the questionnaire found that some believed that individuals should have had previous experience of humanitarian work before undertaking an Ebola mission.

### 4.3 The importance of a professional democratic approach and being a good role model

About half of the participants perceived that they received relevant leadership training for working in an acute epidemic outbreak. They describe it as well organized and superb. The rest felt that it was difficult to get relevant information regarding the leadership structure, because the outbreak was so new and extensive, as expressed in the following quote “The training was basically on management of EVD, there was little to do with leadership and management of teams” (no 23). Sometimes the leadership in the field was also described as unprofessional and ignorant. On the other hand, some nurses had good experiences of supportive and well‐qualified leaders.

When the nurses described their own leadership skills, they experienced them as predominantly satisfactory. They consider themselves as supervisors for the locals: intelligent, flexible, stressful, diplomatic, clear, proud, communicative, humorous, encouraging and solution‐oriented. The importance of being “a good example” was also raised as a fundamental leadership skill. Others were more careful in evaluating their leadership skills and felt that they were not leaders and not ready to take on more responsibility.

On the question of the most appropriate management style, most of the nurses chose democratic. One participant presents this as follows: “A democratic management style. It is very important to collaborate in those kind of missions. Therefore, a good communication and information of staff is mandatory” (no 35). By this, the nurses meant leadership that incorporated listening, professionalism, calmness, experience, structural ability, effectiveness, encouragement, empathy, social competence, support, information and objectivity. The democratic leader in the field should have high morals and see that everyone is working towards the same goal with regard to the local healthcare staff. The participants thought that an authoritarian leader were least suited for an Ebola mission.

### 4.4 The value of timely in‐depth knowledge transfer of experienced former delegates

The last theme relates to knowledge transfer and most participants experienced that the IFRC covered this aspect in an excellent, well‐prepared and sound manner. Although this involved the presentation of a lot of information, it was described as concise and relevant. Several participants appreciated the knowledge transfer that was provided from previous delegates to new ones. However, some said that the knowledge transfer could have been more comprehensive and of higher quality. The improvements proposed by the delegates concern that one could benefit more from former delegates who can serve as support based on their experience. “I would be sending sitreps and encouraging phone calls with returning expats prior to departure” (no 16). In addition, the participants suggested that they wanted to have more practice managing their protective equipment, to have access to site reps and to receive support from the IFRC via Skype and online courses while in the field.

## 5 DISCUSSION

What can be learned regarding health concerns, teamwork, leadership and management and knowledge transfer, based on nurses’ mission in an ETC in West Africa? Overall, it seems that there are some key elements that are of particular importance to the nurses in relation to their mission. Caring for Ebola patients in extreme conditions (Médecins Sans Frontières, 2018a) requires that the delegates are well prepared and receive the support they need. Based on the nurses’ experiences, several different aspects can be taken into account in future missions.

Firstly, a consistent and well‐organized health check and follow‐up of their own health conditions is a prerequisite for delegates to feel safe with their mission. Because Ebola is a lethal virus, it causes worry and anxiety regarding the risk of being infected (Gee & Skovdal, 2017a), which clearly needs to be ruled out at home. This fact also affects mental status, which means that nurses may need extra support in connection with a mission—not least because it has been found that stigmatization towards healthcare workers can be widespread when it comes to Ebola (Gee & Skovdal, 2018). Moreover, relatives should be more informed and involved because they are affected by their loved ones taking on such a demanding mission.

Secondly, the joint preparation before the mission seems to fulfil an important function. Here, it is about developing a common value base and a common approach, where delegates can trust each other. In previous research (cf. Cummings et al., 2009), one can see the importance of this for collaboration to be constructive and to build on teamwork.

A third aspect (that of a professional democratic approach and the ability to be a good role model) seems to be important, as highlighted by Salmela, Koskinen, and Eriksson (2017). Professionalism forms the foundation for successful and sustainable leadership. This includes being a good role model with high moral standards (Tanaka, Taketomi, Yonemitsu, & Kawamoto, 2016) which the nurses appreciated in the field. Finally, the nurses expressed the value of gaining knowledge about their upcoming missions from previous delegates. Knowledge transfer is about collecting and sharing tacit knowledge to make crucial knowledge accessible (Graham et al., 2006).

Lastly, by providing real examples from authentic situations, it seems that former delegates can strengthen those who will face similar situations. The inexperienced nurses find support from those who have more experience in the field, and this at the same time may serve as healing for those who have recently experienced difficult and challenging situations.

Based on the findings of this study, a relevant question is that of how future and advanced nurses can be prepared for disasters as part of the profession. As one of the participants expressed, it probably takes a lot of experience before taking on an Ebola mission. Theoretical knowledge in different subjects is also needed. However, nursing education can contribute to a foundation and an approach that prepares nurses for future disasters both locally and globally. This may be based on global nursing grounded in a humanitarian view where all people's equal value is taken advantage of. The focus of this view is on standing up for vulnerable persons who cannot speak for themselves due to ill health, lack of knowledge and scarce resources. Ebola should not be considered a West African concern but a joint global challenge. How nurses act globally also has consequences for future generations and for the local contexts where they operate. Nurses can contribute to a more solidary and sustainable healthcare system where we take responsibility for each other's health, taking into account the limited resources of the world. Global nursing includes a norm‐critical approach that, in particular, can help to challenge misplaced notions, stereotypes and “truths” about people who have suffered from or being exposed to Ebola (Holmgren, 2017). This helps to counteract the stigma that sometimes occurs among healthcare professionals as well as among the public (Gee & Skovdal, 2018; McGillis Hall & Kashin, 2016).

In summary, we have found that although the nurses generally express that they were satisfied regarding the aspects studied, it also seems that there is potential for improvement to prepare future delegates better. Universities and nursing educators could make even greater use here for future and advanced nurses by contributing with relevant research‐based theoretical and practical knowledge. This would preferably occur in the context of extended further cooperation with the humanitarian organizations that nurses choose to work for during a disaster such as the Ebola outbreak in West Africa.

### 5.1 Study limitations

As in all studies, it is necessary to comment on potential limitations that may have affected the results. Methodologically, it has been a challenge to find nurses to include in the study. Even though the first author was provided with a list of possible informants, it would appear that it was difficult to communicate with them because they had new missions around the world and also because a lack of Internet. It would have been desirable to have a higher response rate. However, in a qualitative study like this, responses from 44 nurses are still acceptable since the focus is on experiences rather than the number of informants. Since the research question was new, we had to design a questionnaire even though it might have been preferable to use a validated existing one. To ensure that the nurses would respond to the aim of the study, a pilot was conducted among nurses with experiences from humanitarian fieldwork. In the process of validating the questions, it was possible to find a consensus regarding the consistency of the questions thanks to the pilot group. Since the data were analysed by the first author, the co‐authors could question and contribute to the interpretation of the result. Based on the systematic procedure and transparency of this study, its findings should be transferable to similar contexts. However, this we leave to the readers to assess. Finally, data could be perceived as out of date, considering the Ebola outbreak occurred a few years ago. However, we believe it is still valuable to gain knowledge about how nurses can be prepared for, supported and followed up after an Ebola mission. This is vital in view of new outbreaks of Ebola and the implications this could have for the Western world's health care.

## 6 CONCLUSION

Most nurses experience the Ebola mission in a positive way. However, when it comes to health concerns, teamwork, leadership and knowledge transfer, they raise some key issues that can be used by humanitarian organizations such as the IFRC to further prepare them as delegates. In future policy‐making, it will be important to focus on what has emerged in this study:

- Personal health management plays an important role in ensuring that the nurses and their families and friends feel safe and secure.
- Pre‐deployment training contributes to a common value base that is necessary for future collaboration.
- A democratic leadership style that incorporates professionalism and good role models is advocated for.
- Nurses express a need for knowledge transfer in a timely manner from experienced previous delegates.

With the risk of further Ebola outbreaks, the care of Ebola patients in a solidary and sustainable manner is a continuing concern. To be able to achieve this, there is a need for further research from a global nursing perspective to counteract notions and stereotypes regarding healthcare workers who care for vulnerable people suffering from Ebola.

## CONFLICT OF INTEREST

No conflict of interest exists regarding the present study.

## AUTHOR CONTRIBUTIONS

J Holmgren was involved in the design, data analysis and preparation of the present manuscript. S Paillard‐Borg, E von Strauss and P Saaristo were involved in the design and data collection. All authors contributed with critical revision of the manuscript before submitting it.

## APPENDIX A

### Global nursing in acute viral epidemic outbreaks: Before, during and after deployment

A joint research project between The Swedish Red Cross University College (SRCUC) and The International Federation of Red Cross and Red Crescent Societies (IFRC).

Please write your answers below each question, or if you use a separate paper, make sure to include the corresponding question number next to your answer for identification purposes.

**1. Background information**

- How old are you?
- What is your gender?
- In which country do you reside?
- How were you recruited for this deployment; via the RC ERU or from another source?
- Did you have any previous RC ERU training(s) before this deployment?
- Do you have any previous humanitarian work experience?
- How many times have you been deployed at the Ebola Treatment Centre (ETC) in Kenema?
- When did your deployment at the ETC end (Write which month and year. If more than once, please provide the last)?

**2. Health concerns**

- Did you find that your personal health preparation by the RC was adequate?
  If yes, why was it adequate?
  If no, why not?
- Did you find that your personal health follow up by the RC was adequate?
  If yes, why was it adequate?
  If no, why not?
- Did your relatives/friends/employers/colleagues receive adequate information and an opportunity to voice their concerns before, during and after your deployment?
- What improvements would you suggest to the RC in regard to personal health management?

**3. Handling stress**

- How do you cope and handle stress? (i.e., dealing with traumatic events, risk of contamination, operating in draining environments and people's attitudes towards you when returning home.)
  Pre, during and after deployment. Please give examples.
- How could the RC improve its support for stress management to medical staff working in this type of operation?

**4. Socio‐cultural environment**

How did you adapt your work in an environment with different understanding of health and cause of illness and relating to traditional healers and priests?

**5. Teamwork, principles and behaviour**

- How do you consider the relevance of the pre‐deployment training on teamwork, principles and behaviour?
- What are the changes you would suggest the pre‐deployment training to make for your work to become more effective and relevant?

**6. Leadership and management**

- Did the RC provide adequate leadership training for working in an acute epidemic outbreak?
  If yes, what was adequate?
  If no, what was not?
- How would you describe your leadership skills in acute epidemic outbreaks?
- What management styles do you consider most appropriate for this type of operation?

**7. Knowledge transfer**

- How do you consider the pre‐deployment knowledge transfer (sufficiently deep, wide, relevant)?
- If you were to plan and organize the pre‐deployment knowledge, which changes would you implement?

**8. Attitudes**

What kind of reactions have you met in your daily environment when returning home after having worked in an acute epidemic outbreak?

**9. Conclusions**

How would you consider, in general, your preparation and training by the RC compared with health workers working for other humanitarian organizations?

**10. Miscellaneous**

Please feel free to add any comments you would like.
